# DIOL Triterpenes Block Profibrotic Effects of Angiotensin II and Protect from Cardiac Hypertrophy

**DOI:** 10.1371/journal.pone.0041545

**Published:** 2012-07-23

**Authors:** Ruben Martín, Maria Miana, Raquel Jurado-López, Ernesto Martínez-Martínez, Nieves Gómez-Hurtado, Carmen Delgado, Maria Visitación Bartolomé, José Alberto San Román, Claudia Cordova, Vicente Lahera, Maria Luisa Nieto, Victoria Cachofeiro

**Affiliations:** 1 Departamento de Fisiología, Facultad de Medicina, Universidad Complutense, Madrid, Spain; 2 Instituto de Ciencias del Corazón (ICICOR), Hospital Clínico, Valladolid, Spain; 3 Departamento de Farmacología, Facultad de Medicina. Universidad Complutense, Madrid, Spain; 4 Centro de Investigaciones Biológicas, Consejo Superior de Investigaciones Científicas (CSIC), Madrid, Spain; 5 Departamento de Oftalmología y Otorrinolaringología, Facultad de Psicología, Universidad Complutense, Madrid, Spain; 6 Instituto Biología y Genética Molecular, CSIC-UVA, Valladolid, Spain; Universidade Federal do Rio de Janeiro, Brazil

## Abstract

**Background:**

The natural triterpenes, erythrodiol and uvaol, exert anti-inflammatory, vasorelaxing and anti-proliferative effects. Angiotensin II is a well-known profibrotic and proliferative agent that participates in the cardiac remodeling associated with different pathological situations through the stimulation and proliferation of cardiac fibroblasts. Therefore, the aim of the study was to investigate the preventive effects of the natural triterpenes erythrodiol and uvaol on the proliferation and collagen production induced by angiotensin II in cardiac myofibroblasts. Their actions on cardiac hypertrophy triggered by angiotensin II were also studied.

**Methodology/Principal Findings:**

The effect of erythrodiol and uvaol on angiotensin II-induced proliferation was evaluated in cardiac myofibroblasts from adult rats in the presence or the absence of the inhibitors of PPAR-γ, GW9662 or JNK, SP600125. The effect on collagen levels induced by angiotensin II was evaluated in cardiac myofibroblasts and mouse heart. The presence of low doses of both triterpenes reduced the proliferation of cardiac myofibroblasts induced by angiotensin II. Pretreatment with GW9662 reversed the effect elicited by both triterpenes while SP600125 did not modify it. Both triterpenes at high doses produced an increase in annexing-V binding in the presence or absence of angiotensin II, which was reduced by either SP600125 or GW9662. Erythrodiol and uvaol decreased collagen I and galectin 3 levels induced by angiotensin II in cardiac myofribroblasts. Finally, cardiac hypertrophy, ventricular remodeling, fibrosis, and increases in myocyte area and brain natriuretic peptide levels observed in angiotensin II-infused mice were reduced in triterpene-treated animals.

**Conclusions/Significance:**

Erythrodiol and uvaol reduce cardiac hypertrophy and left ventricle remodeling induced by angiotensin II in mice by diminishing fibrosis and myocyte area. They also modulate growth and survival of cardiac myofibroblasts. They inhibit the angiotensin II-induced proliferation in a PPAR-γ-dependent manner, while at high doses they activate pathways of programmed cell death that are dependent on JNK and PPAR-γ.

## Introduction

Cardiac fibroblasts are one of the major cellular components of the heart. They play an important role in the maintenance of structural integrity and normal cardiac function, where both cell-cell and cell-extracellular matrix interactions are essential [1], [2]. They participate in the reparative response of damaged tissue to wound healing, not only through controlled extracellular matrix production, but also through proliferation, migration and differentiation into hypersecretory myofibroblasts [3]–[5]. The acquisition of smooth-muscle-like properties in fibroblasts is associated with exacerbation of extracellular matrix production [6], which can trigger impairment of cardiac function by facilitating reduced contractibility and arrhythmias, and which then ultimately contribute to heart failure [7]–[9]. The activation of cardiac fibroblasts to myofibroblasts is greatly enhanced in chronic cardiac diseases and after acute cardiac events [9]–[11]. This transformation is controlled by a variety of stimuli, including growth and vasoactive factors such as angiotensin II, cytokines and mechanical stimuli [12].

Angiotensin II plays a central role in the development and complications of cardiovascular diseases by exerting, among other types of action, a fibrotic one [13]–[15]. This participation has been demonstrated by the effectiveness of drugs that interact with this system on patients with left ventricular hypertrophy or heart failure [15]. Its fibrotic action involves the activation not only of growth factors such as connective tissue growth factor (CTGF) but also new mediators such as galectin 3, which is associated with adverse long-term cardiovascular outcomes in patient with heart failure [16], [17].

The Mediterranean diet, in which olive oil is the major source of dietary fat intake, has been associated with low incidence of cardiovascular diseases [18], [19] and cancer [20]–[22]. Although these health benefits have long been attributed to a high content of monounsaturated fatty acids (oleic acid), a wide variety of minor components are under evaluation. Among these bioactive compounds are the triterpenes including the diols, uvaol and erythrodiol [23]. Many pharmacological properties, including antiinflammatory, antitumoral and antioxidant activities [24]–[26], have been reported for these compounds. In addition, recent studies have suggested beneficial effects on the cardiovascular system, since antihypertensive vasodepressor, cardiotonic, and antidysrhythmic properties have been reported [27]–[29]. However, the effect of these compounds on normal cells, especially on cardiac cells, is unknown. Thus, in the search for novel pharmacological approaches for the management of cardiovascular pathologies, the antiproliferative and antifibrotic effects of these triterpenes are noteworthy. We thus proposed to investigate *in vivo* and *in vitro* the potential benefits of erythrodiol and its isomer, the ursane diol uvaol, on cardiac effects of angiotensin II. To this end, we explore their modulatory effects on angiotensin II-induced proliferation and collagen production in cardiac myofibroblasts as well as the possible mediators involved. In addition, we explore the effect of erythrodiol and uvaol on the cardiac hypertrophy induced by angiotensin II in mice.

## Methods and Materials

### Ethics Statement

The Animal Care and Use Committee of Universidad Complutense of Madrid and Universidad de Valladolid approved all experimental procedures according to guidelines for ethical care of experimental animals of the European Community.

### Animals

Twenty four 8-week-old C57BL/6J mice (Harlan Ibérica, Barcelona, Spain) were randomly divided into 4 groups of 6 animals. Angiotensin II (Sigma) was administered with osmotic mini-pumps (Alzet model 1002, 1.44 mg Kg^−1^ day^−1^) for 2 weeks. Some of the animals were treated for the same period with erythrodiol or uvaol at a dose of (50 mg Kg^−1^ day^−1^) by i.p. injection. In the control group, mice received vehicle (saline solution) for 2 weeks. The dosage of angiotensin II, erythrodiol and uvaol were chosen from previous studies. In the case of angiotensin II, this dose induced left ventricle hypertrophy and fibrosis in mice [30]; in the case of both triterpenes, treatment was able to prevent the development of multiple sclerosis in mice [25].

### Isolation of Cardiac Fibroblast

**Figure 1:**
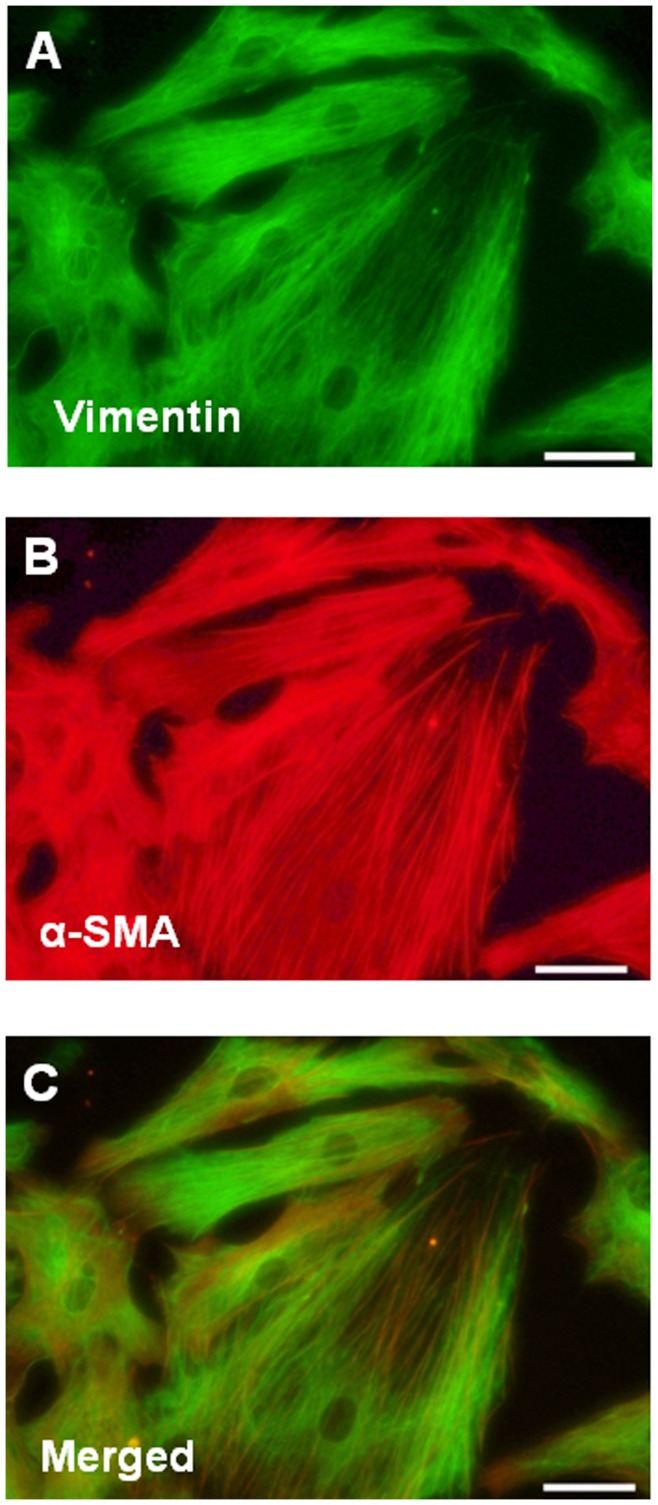
Representative immunocytochemistry images of cardiac myofibroblasts examined by fluorescence microscopy. Vimentin staining (A), α-smooth muscle actin (α-SMA) staining (B) and vimentin and α-SMA staining merged (C). Magnification 40X. Scale bar: 50 µm.

Non-myocytes from adult male Wistar rats (Harlan Ibérica, Barcelona, Spaina) weighing 250–300 g were obtained by differential centrifugation of cardiac cells released after retrograde Langendorf perfusion and enzymatic digestion of the hearts, as previously described [31]. Briefly, rats were anesthetized with sodium pentobarbital (50 mg/kg) before the heart was removed. Afterwards, hearts were first perfused for 2–3 min at 36–37°C with a nominally calcium-free Tyrode solution containing 0.2 mM EGTA, and then for approximately 3–4 min, with the same Tyrode solution containing 251 UI of collagenase type II (Worthington) and 0.1 mM CaCl_2_. At the end of the perfusion period, the heart was removed from the Langendorff apparatus and cut off, chopped into small pieces and gently stirred in a solution containing 1 mg/ml of bovine serum albumin (BSA, Sigma). The fibroblasts were collected by centrifugation and resuspended in DMEM. The homogeneity of these primary isolates was assessed by immunochemistry using anti-vimentin (Novocastra Laboratories, Newcastle, UK). Cells in the present study were used at 2–3 passages. Characterization of the cells using immunocytochemistry revealed consistent coexpression of vimentin (Figure 1A) and α-smooth muscle cell actin (α-SMA; Figure 1B) and a consistent coexpression of both antigens (Figure 1C) through passage 1, indicating that the cells possessed a myofibroblasts phenotype.

### Cell Culture Conditions

The cells were maintained in DMEM medium supplemented with 10% FBS, 10 mM L-glutamine, 100 U/ml penicillin/streptomycin, 10 mM, L pyruvate and 2 mM HEPES. The cells were seeded at a density of 0.5×10^6^ cells in a T-175 tissue culture flask and then grown as monolayer culture. Cells were passaged with 0.25% trypsin in 0.01% EDTA whenever they became confluent. All assays in the present study were done at temperatures of 37°C, 95% sterile air and 5% CO_2_ in a saturation humidified incubator.

### Cell Proliferation Assay

Cell proliferation was assessed using the CellTiter 96 Non-Radioactive Cell Proliferation Assay (Promega Corporation, Madison, WI, USA). Cardiac myofibroblasts were seeded on 96-well plates (20×10^3^cells/well) in DMEM medium and were allowed to attach for 24–36 hours. Afterwards, cells were switched to serum-free medium for 24-h. Cells were then treated with angiotensin II in the presence or absence of different doses of (0.5–50 µM) erythrodiol or uvaol for 24 hours. Cells were pretreated with either vehicle or triterpenes for 30 min before the addition of angiotensin II. The proliferative response was quantified by adding MTT tetrazolium solution (20 µl/well). After 2–3 hours of incubation absorbance was measured at 490 nm in a microplate reader (ASYS Hitech GmbH, Austria). Three different assays were each performed in quintuplicate.

In some experiments, cells were pretreated for 30 min with either the selective PPAR-γ antagonist (2-chloro-5-nitro-N-phenylbenzamide; GW9662: 1–10 µM), mitogen-activated protein kinase (MEK) inhibitor (PD98059; 5–50 µM) or stress-activated c-Jun N-terminal kinase (JNK) inhibitor (SP600125; 5–20 µM).

### Analysis of Apoptosis

After a 24-hour treatment with 1 µM of angiotensin II in the presence or absence of different doses of erythrodiol or uvaol, cells were used for an Annexin V-PE Apoptosis Assay, as previously described [23]. Briefly, 1×10^5^ cells were resuspended in 0.5 ml binding buffer (10 mM HEPES, pH 7.4, 150 mM NaCl, 2.5 mM CaCl_2_, 1 mM MgCl_2_, 4% BSA), and incubated for 15 min with 2.5 ng/ml Annexin V-PE, followed by flow cytometric analysis using an EPICS XL cytofluorometer, Beckman-Coulter. In some experiments, cardiac myofibroblasts were cultured in the presence of the inhibitors, PD98059, SP600125, or GW9662 in order to evaluate the participation of ERK, JNK and PPAR-γ pathways in the apoptotic effect induced by the triterpenes.

### Western Blot

Total and nuclear proteins were prepared as previously described from cell extracts isolated from cardiac myofibroblasts treated with either 1 µM angiotensin II or vehicle for 12 hours with or without 5 µM of triterpenes. Proteins were separated by SDS-PAGED on 10% polyacrylamide gels and transferred to polyvinylidene difluoride membranes (Hybond-P; Amersham Biosciences, Piscataway, NJ). Membranes were probed with primary antibody for α-SMA (Biocare Medical, CA, USA), PPAR-γ (Santa Cruz, Inc, USA), collagen I (AbD Serotec, Oxford, UK), connective tissue growth factor (CTGF; Torrey Pines Biolabs Inc, East Orange, NJ) and galectin 3 (Thermo Scientific, Rockford, IL) followed by incubation with an HRP-linked secondary antibody. Signals were detected using the ECL system (Amersham Pharmacia Biotech). Results are expressed as an n-fold increase over the values of the control group in densitometric arbitrary units. Phosphorylated ERK1/2 (Cell Signaling Technology, Inc, New England, USA) and total ERK1/2 (Zymed Laboratories, CA, USA) protein levels were analyzed in cell extracts isolated from cardiac myofibroblasts treated with 1 µM of angiotensin II or vehicle for 15 minutes with or without different doses of the triterpenes.

### Morphological and Histological Evaluation

Hearts were arrested in diastole using KCl before harvesting, and then dehydrated and embedded in paraffin. Histological determinations in cardiac tissue were performed in 4 µm-thick sections. In Masson’s trichrome stained sections, left ventricular cross sectional area (LVCSA) and left ventricular wall thickness (LVWT) were measured. Two or three serial sections for each animal at the midregion area were analysed with a 5X objective lens under microscopy transmitted light. For cardiomyocyte cross sectional area, at least 60–80 cardiomyocytes per animal were measured with a 40X objective lens under microscopy transmitted ligh. Cardiomyocyte with visible nucleus and intact cellular membrane were only measured.

Fibrosis was quantified in Picro-sirius red-stained sections. The area of interstitial fibrosis was identified after excluding the vessel area from the region of interest, as the ratio of interstitial fibrosis or collagen deposition to the total tissue area. For each sample, 10 to 15 fields were analyzed with a 40X objective lens under microscopy transmitted light. Perivascular fibrosis was also analysed. All measurements were performed blind in an automated image analysis system (Metamorph 7.0, Molecular Devices Corporation, USA). Images were calibrated with known standards. A single researcher unaware of the experimental groups performed the analysis.

### Immunocytochemistry

Cardiac myofibroblasts were fixed in 4% paraformaldehyde for 30 min and permeabilized with 1% Triton ×−100. Preincubation was carried out for 30 min in a PBS solution containing 30% normal horse serum. Cells were then incubated overnight at 4°C in a solution containing 1/100 anti-vimentin (Novocastra, Leyca Byosystems, Newcastle, UK) or 1/100 anti-α-SMA monoclonal antibodies (Oncogene Biocare medical, Concord, CA). After three washings (5 min each) in PBS, the cells were incubated for 1 h in fluorescein or Texas red horse anti-mouse IgG (Vectastin Vector) 1/200 in PBS [32]. Nuclei were stained with DAPI (Sigma-Aldrich, Germany). Negative controls were carried out. Images were visualized and photographed with a 40X objective in a Leica DMI 3000 B microscopy.

### Evaluation of Brain Natriuretic Peptide (BNP) by an Enzyme-Linked Immunosorbent Assay (ELISA)

BNP levels were determined in serum samples by using a mouse BNP-specific ELISA (RayBiotech, Norcross, GA, USA) according to the manufacturer’s protocols. Data were processed and expressed as concentration of BNP/ml of serum samples.

### Statistical Analysis

Data are expressed as mean ± SEM. Cell proliferation data are expressed as the percentage of the values in control conditions. Data were analyzed using a one-way analysis of variance, followed by a Newman-Keuls or Dunnet test to assess specific differences among doses or control conditions, respectively using GraphPad Software Inc. (San Diego, CA, USA). The predetermined significance level was p<0.05.

## Results

### Erythrodiol and Uvaol Modulate the Proliferative Response of Angiotensin II

**Figure 2:**
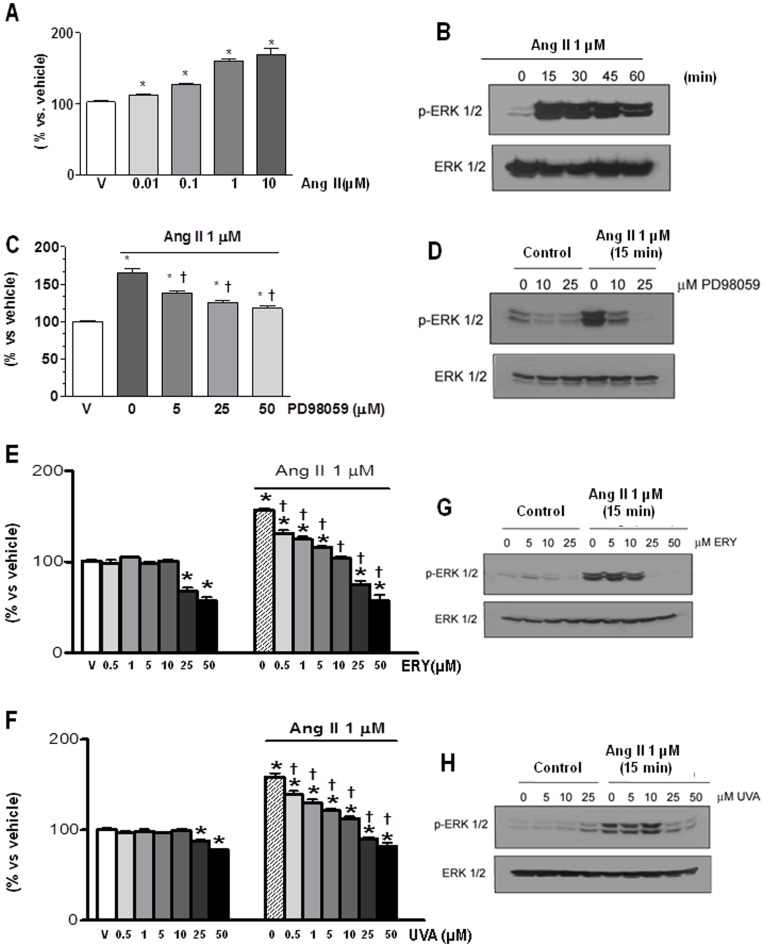
Effect of erythrodiol or uvaol on the proliferation and ERK 1/2 phosphorylation induced by angiotensin II. Cardiac myofibroblasts were stimulated with different doses of angiotensin II (Ang II; A) or with 1 µM of Ang II at different times (B). Cells in the absence or presence of different concentrations of a specific MEK inhibitor (PD98059; C and D), erythrodiol (ERY; E and G) or uvaol (UVA; F and H). After 24 h of incubation at 37°C (A, C, E and F), cell proliferation was determined by an MTT assay and expressed as percent of controls (vehicle, v). (B, D, G and H), after 15 min stimulation, whole cell lysates were extracted and protein phosphorylation was assessed by Western blotting using phospho-ERK and total ERK antibodies. Membranes always were stained with Ponceau S as a loading control. Representative immunoblots of 3 experiments. Values are mean ± SEM of three assays; *p<0.05 *vs* vehicle (V). †p<0.05 vs either 0 (absence of ERY or UVA) or Ang II.

Effects of erythrodiol and uvaol were examined on cardiac myofibroblasts proliferation induced by angiotensin II. As previously described, angiotensin II induced a dose-dependent increase in cardiac myofibroblasts growth (Figure 2A). The dose inducing the maximal change, 1 µM, was used for all subsequent experiments. Angiotensin II treatment also triggered, as expected, a strong and sustained activation/phosphorylation of ERK1/2 (Figure 2B), which play a central role in the regulation of myofibroblast proliferation. As shown in Figure 2C and D the presence of the MEK inhibitor PD98059 strongly reduce the proliferative effect and abrogated ERK phosphorylation and induced by angiotensin II (1 µM).

The presence of either erythrodiol or uvaol (Figures 2E and 2F respectively) was able to reduce the angiotensin II-induced proliferation in a dose-dependent manner. Interestingly, independently of the presence or not of angiotensin II, the highest doses of both triterpenes (25–50 µM) seems to influence cell viability, because they were able to reduce the number of cells to levels lower than those of basal conditions. At such doses, both triterpenes reduced the phosphorylation of ERK1/2 (Figures 2G and 2H, respectively). No effect on ERK 1/2 phosphorylation was observed with the lower doses of either triterpene (5–10 µM).

**Figure 3:**
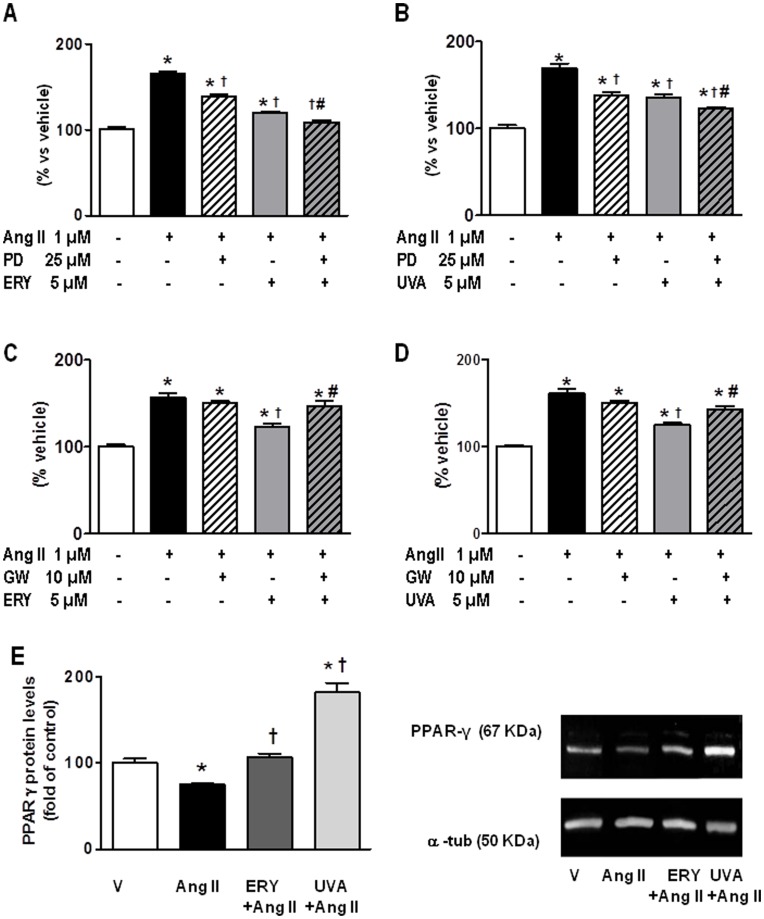
Effect of specific inhibitors on the antiproliferative activity of erythrodiol or uvaol in angiotensin II-treated cardiac myofibroblasts. Cells pretreated for 30 min with the specific inhibitors of either MEK (A and B; PD98059) or PPAR-γ (C and D; GW9662) were stimulated with 1 µM of angiotensin II (Ang II) for 24 hours in the presence of 5 µM of either erythrodiol (ERY; A and C) or uvaol (UVA; B and D). Proliferation was determined by an MTT assay. Data are expressed as percent of unstimulated cells. Values are mean ± SEM of three assays. Panel E represents the effect of either ERY (5 µM) or UVA (5 µM) on nuclear PPAR-γ protein levels in Ang II-treated cardiac myofibroblasts. Nuclear proteins from cells stimulated with 1 µM of Ang II from 12 hours in the presence or absence of the indicated triterpene were analysed by western blotting a specific antibody against PPAR-γ. Representative immunoblots of 4 experiments. Quantification of band intensities was measured by densitometry and normalized to respective α-tubulin. *p<0.05 *vs* vehicle. †p<0.05 vs angiotensin II. #p<0.05 vs erythrodiol or uvaol.

The presence of PD98059 (25 µM) in cells pretreated with either erythrodiol or uvaol at 5 µM, further reduced the proliferation induced by angiotensin II (Figures 3A and 3B, respectively). Next, in order to explore the role of PPAR-γ on the antiproliferative effects of erytrodiol and uvaol, we used the specific PPAR-γ inhibitor, GW9662 (10 µM). As shown in Figures 3C and 3D, pretreament with the specific PPAR-γ inhibitor, GW9662, reversed the reduction in angiotensin II-induced proliferation of cardiac myofibroblasts observed in the presence of either erythrodiol (5 µM) or uvaol (5 µM). In addition, the reduced nuclear protein levels of PPAR-γ observed in angiotensin II-treated cells were reversed in those pretreated with both triterpenes (Figure 3E).

### Erythrodiol and Uvaol at High doses Induced Apoptosis of Cardiac Myofibroblasts

**Figure 4:**
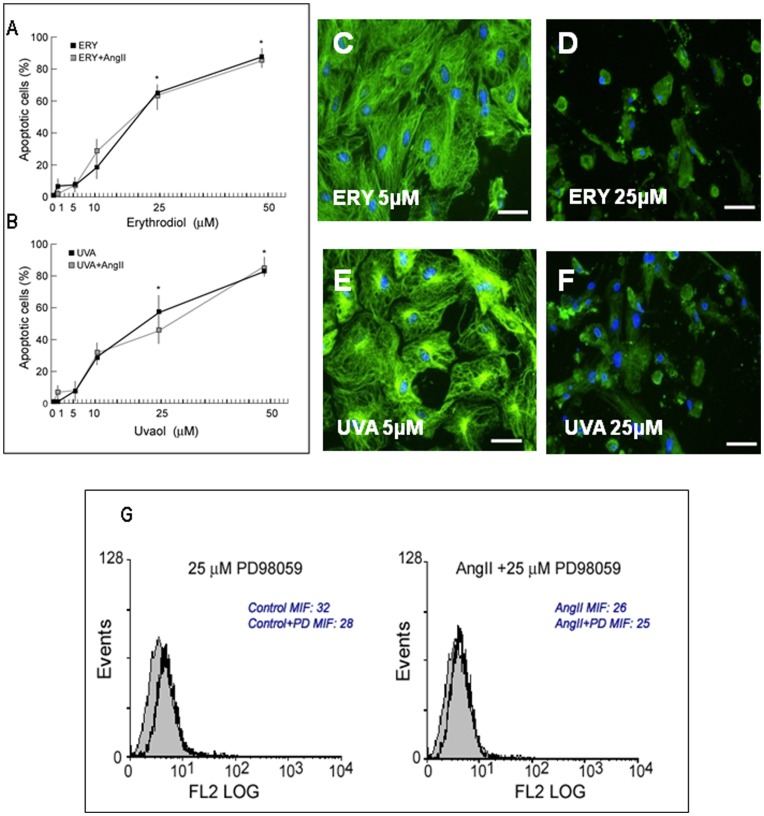
Efect of erytrodiol and uvaol in cardiac myofibroblasts apoptosis. Cardiac myofibroblast were treated with different doses of erythrodiol (ERY; A) or uvaol (UVA; B) in the presence or absence of angiotensin II (Ang II, 1 µM). After 24 h of stimulation, cells were labeled with annexin-V PE and analyzed by flow cytometry. Values are mean ± SEM of three experiments. *p<0.05 vs cells in the absence of either erythrodiol or uvaol. Figures C–F show representative immunocytochemistry images of cardiac myofibroblasts treated for 24 hours with erythrodiol (C: 5 µM; D: 25 µM) or uvaol (E: 5 µM; F: 25 µM) examined by fluorescence microscopy. Vimentin staining is shown in green and nuclei staining in blue. Magnification 40X. Scale bar: 50 µm. Figure G: Effect of a specific inhibitor of MEK (PD9805; 25 µM) on the apoptosis in the presence or absence of angiotensin II in cardiac myofibroblasts. Cells obtained after PD98059 treatment in the absence of the inhibitor (open black curve) are compared with cells treated in the presence of the inhibitor (open gray curves). Solid gray curves represent resting control cells.

To determine whether the anti-proliferative effects of erythrodiol and uvaol were associated with the beginning of apoptotic processes in cardiac myofibroblasts, we monitored, as an apoptotic feature, the appearance of phosphatidylserine on the cell surface using an annexin-V binding assay. Minimal differences in annexin V–positive cells, which were not statistically significant (p>0.05), were found at the lower doses (1–10 µM) in the erythrodiol- or uvaol-stimulated cells after 24 hours of culture, as compared with unstimulated ones (Figures 4A and 4B, respectively). Only the highest doses of either erythrodiol or uvaol (25–50 µM) were able to induce a significant increase in cells stained positive with annexin V–phycoerythrin (independently or not of the presence of angiotensin II) when compared to the unstimulated cells (p<0.05); this suggests that these high doses of both triterpenes are able to induce apoptosis of cardiac myofibroblasts (Figure 4A and 4B, respectively). As shown in Figures 4C, 4D, 4F, either erythrodiol or uvaol at the dose of 25 µM (Figures 4D and 4F, respectively) cause retraction, rounding and shrinking of cardiac myofibroblasts and vimentin filament rearrangement; this was not observed at the dose of 5 µM (Figures 4C and 4E, respectively), where cells presented an appearance similar to that of control cells (Figure 1).

To determine if apoptosis of cardiac myofibroblasts was mediated by the abrogated phosphorylation of ERK 1/2, the apoptotic response was measured in cells pretreated with the inhibitor PD98059 either in the presence or absence of angiotensin II. No differences in the mean fluorescence intensity of annexin V incorporation were observed in cells exposed to 25 µM of PD98058, a dose that effectively prevents ERK1/2 phosphorylation, compared to untreated ones (Figure 4G).

**Figure 5:**
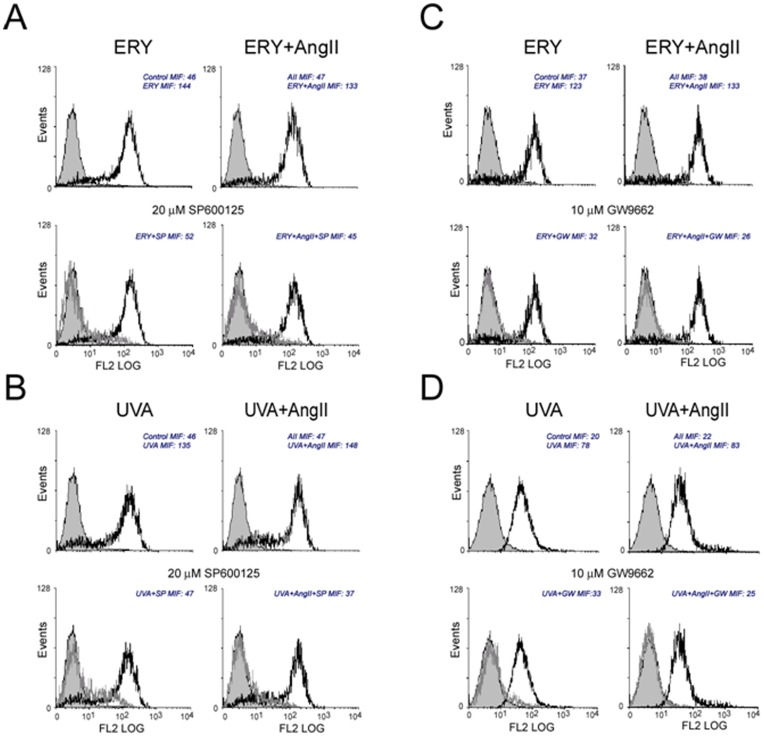
Effect of specific inhibitors on the apoptotic activity of erythrodiol or uvaol in cardiac myofibroblasts. Effect of a specific inhibitor of either JNK (SP600125) or PPAR-γ, (GW9662) on the apoptosis induced by either erythrodiol (ERY; 25 µM A and C, respectively) or uvaol (UVA; 25 µM; B and D, respectively) in the presence or absence of angiotensin II (Ang II; 1 µM) in cardiac myofibroblasts. Representative of 3 experiments. In all panels, cells obtained after triterpene treatment in the absence of the inhibitor (open black curve) are compared with cells treated in the presence of the inhibitor (open gray curves). Solid gray curves represent control cells.

In contrast, triterpene-induced cell death was markedly reversed by pretreatment with 20 µM of the specific JNK inhibitor, SP600125 (Figures 5A, and 5B). The same protective effect of the inhibitor SP600125 was observed when cells were stimulated simultaneously with the triterpenes and angiotensin II. Cellular viability in control cardiac myofibroblast was not affected by the inhibitor at the tested dose. The presence of the p38 inhibitor SB203580 did not modify the apoptotic response triggered by the triterpenes (data not shown).

Finally, the role of PPAR-γ was evaluated by conducting annexin-V binding experiments in cells pre-treated with different doses of the PPAR-γ antagonist GW9662. As shown in figures 5C and 5D, the apoptotic response in erythrodiol- and uvaol-treated cells was inhibited by the presence of the inhibitor GW9662 (10 µM), suggesting that this response was PPAR-γ-dependent. The inhibitory effect of GW9662 was also observed in cells exposed simultaneously to triterpene and angiotensin II.

### Erythrodiol and Uvaol Abrogate the Fibrotic Effect of Angiotensin II on Cardiac Myofibroblasts

**Figure 6:**
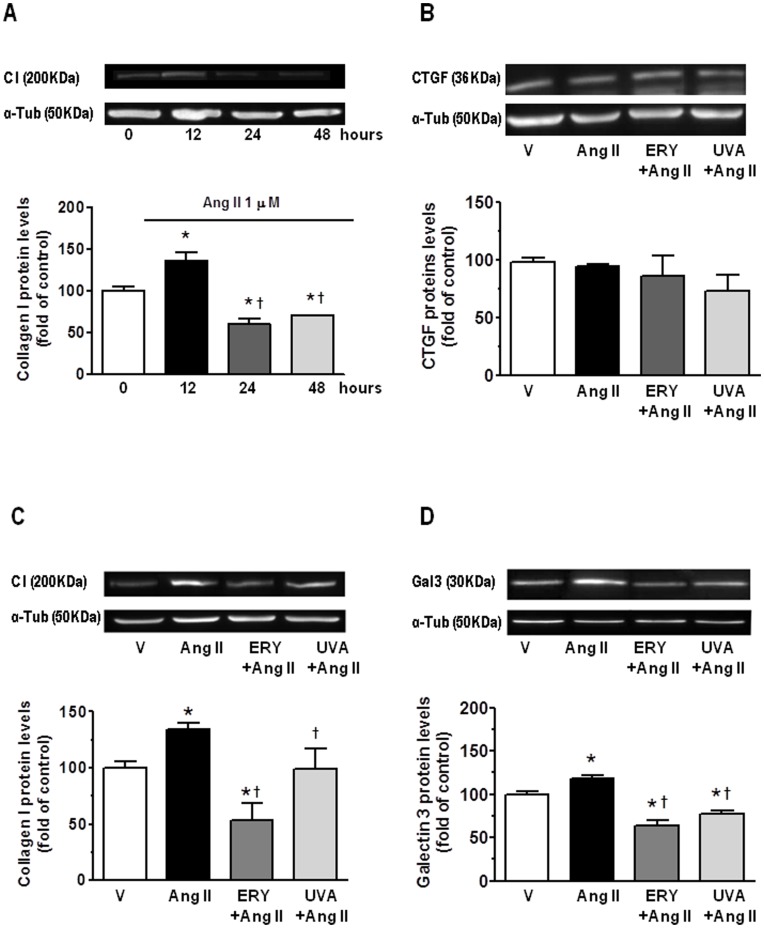
Effect of erythrodiol and uvaol on the fibrotic effect of angiotensin II in cardiac myofibroblasts. Time course of angiotensin II (Ang II; 1 µM)-stimulated collagen I protein production (A). Effect of erythrodiol (ERY; 5 µM) or uvaol (UVA; 5 µM) on CTGF (B), collagen I (C) and galectin 3 (D) protein expression in angiotensin II-treated cardiac myofibroblasts for 12 hours. Representative immunoblots of 4 experiments. Values are mean ± SEM of four assays. *p<0.05 *vs* vehicle. †p<0.05 vs angiotensin II. Quantification of band intensities was measured by densitometry and normalized to respective α-tubulin.

Angiotensin II at the dose of 1 µM induced an increase in collagen I synthesis in cardiac myofibroblasts (Figure 6A), reaching the maximum effect at 12 hours. This production seems to be independent of CTGF because no changes in protein expression were observed at the time angiotensin II induced the maximal synthesis of collagen I (Figure 6B). To determine whether these triterpenes were able to modify angiotensin II-induced profibrotic effects, cardiac myofibroblasts were exposed to erythrodiol (5 µM) or uvaol (5 µM). Their presence (Figure 6C) was able to reduce collagen I production induced by angiotensin II (1 µM). However, no changes were observed in cells pretreated with the triterpenes in absence of angiotensin II (data not shown). The increase in galectin 3 protein levels induced by angiotensin II was smaller in the presence of either erythrodiol (5 µM) or uvaol (5 µM) (Figure 6D). However, no changes were observed in galectin 3 protein levels in cardiac myofibroblasts pretreated with the triterpenes in absence of angiotensin II (data not shown).

### Erythrodiol and Uvaol Abrogate the Fibrotic Effect of Angiotensin II on Mice

**Figure 7:**
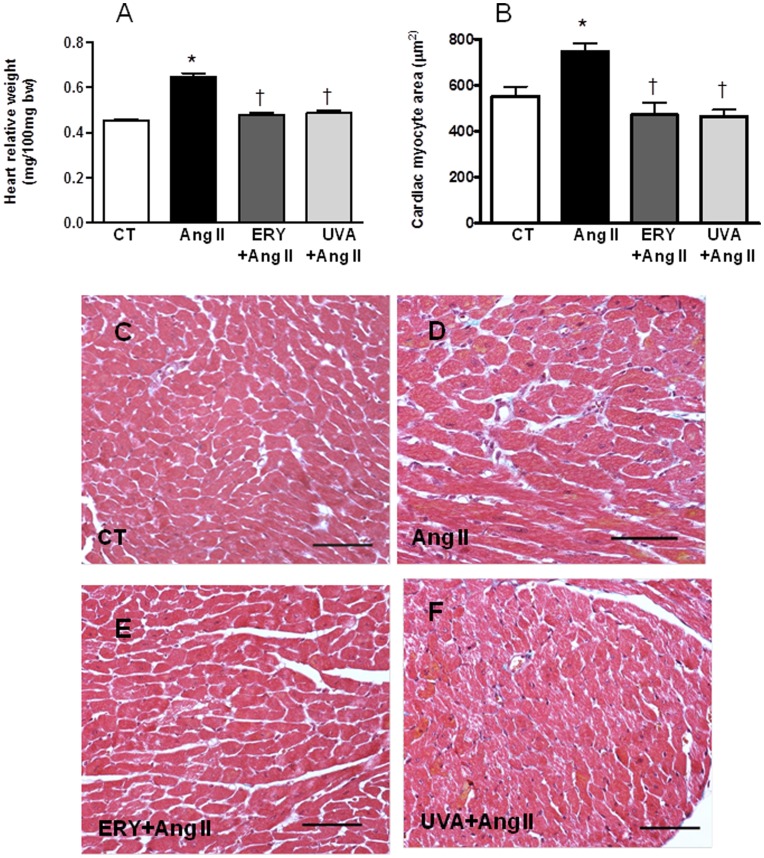
Effect of erythrodiol and uvaol on the hypertrophyc effects of angiotensin II on mice. Mice infused with angiotensin II (Ang II; 1.44 mg Kg^−1^ day ^−1^) were treated with erythrodiol (ERY; 50 mg Kg^−1^ day^−1^) or uvaol (UVA; 50 mg Kg^−1^ day ^−1^) for two weeks. (A) relative heart weight and (B) cardiac myocyte area. Representative microphotographs of myocardial sections from control (C, CT), angiotensin II-infused animals treated with vehicle (D), erythrodiol (E), or uvaol (F). Magnification 40X. Samples were stained with Masson’s trichrome technique. Scale bar: 100 µm. Values are mean ± SEM of 5–6 animals. *p<0.05 *vs* control. †p<0.05 vs angiotensin II.

**Figure 8:**
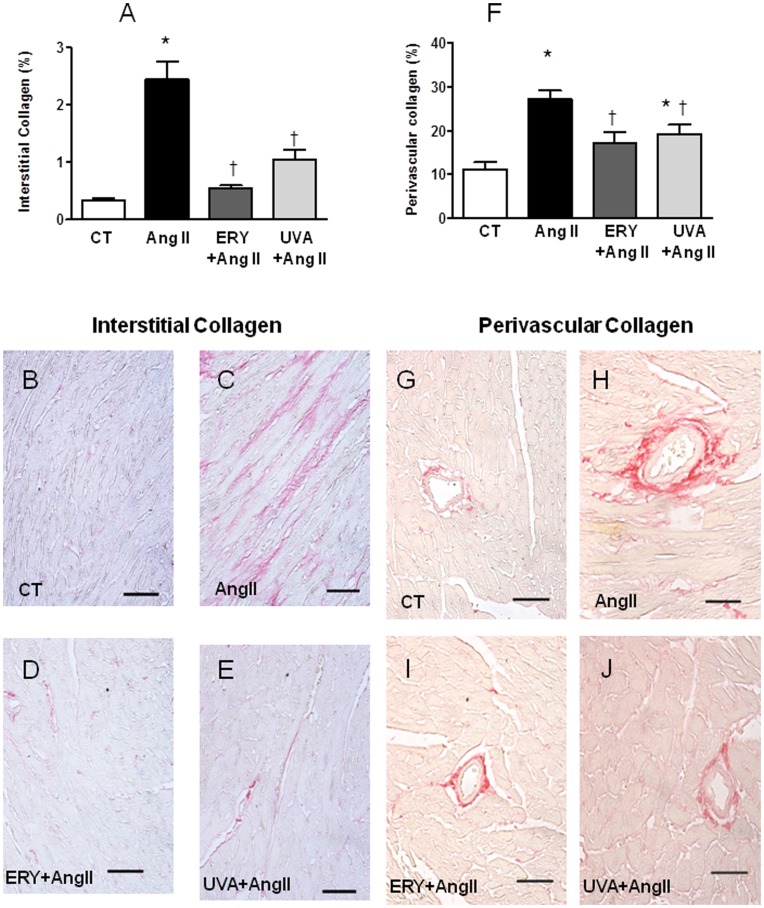
Effect of erythrodiol and uvaol on the fibrotic effects of angiotensin II on mice. Mice infused with angiotensin II (Ang II; 1.44 mg Kg^−1^ day^−1^) were treated with erythrodiol (ERY; 50 mg Kg^−1^ day^−1^) or uvaol UVA (50 mg Kg^−1^ day^−1^) for two weeks. Fibrosis was assessed by Picro-sirius red staining procedure. Interstitial (A) and perivascular (F) collagen quantification. Representative microphotographs of myocardial sections showing interstitial and perivascular fibrosis from control (B, G), angiotensin II-infused animals treated with vehicle (C, H), erythrodiol (D, I), or uvaol (E, J). Magnification 40X. Scale bar: 100 µm. Values are mean ± SEM of 5–6 animals. *p<0.05 *vs* control. †p<0.05 vs angiotensin II.

**Figure 9:**
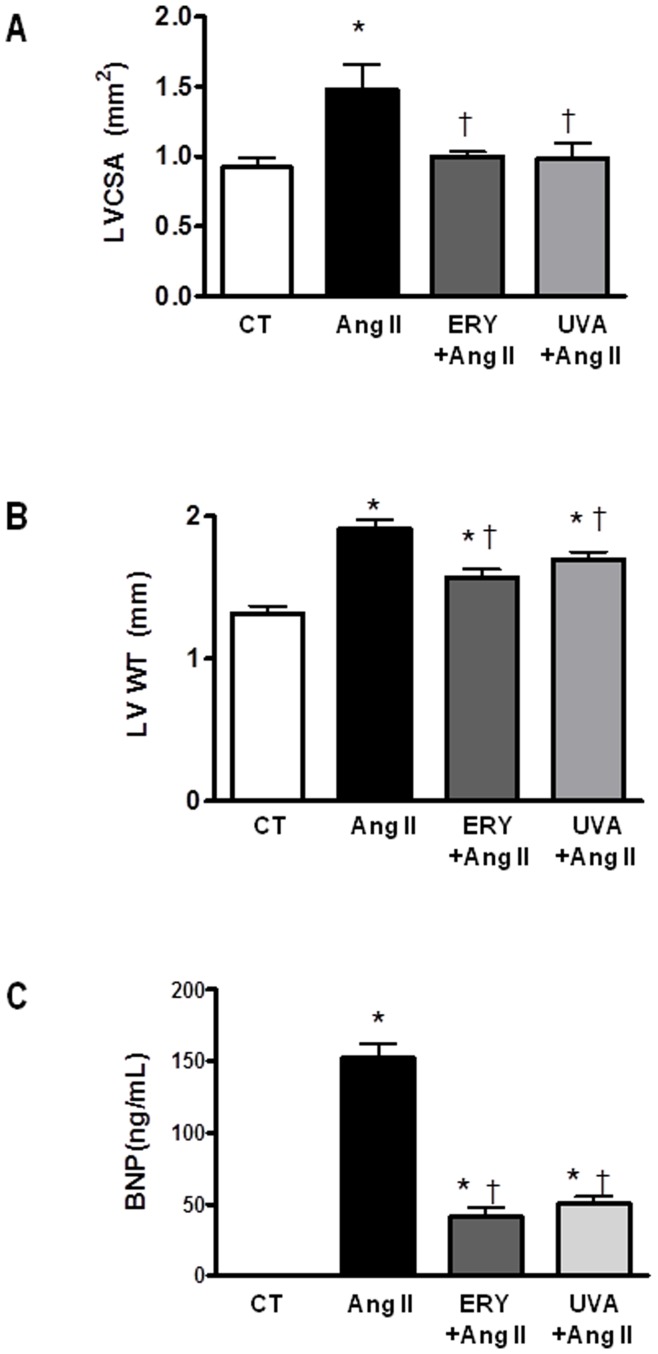
Effect of erythrodiol and uvaol on and left ventricle remodeling and serum BNP levels induced by angiotensin II on mice. Mice infused with angiotensin II (Ang II; 1.44 mg Kg^−1^ day^−1^) were treated with erythrodiol (ERY; 50 mg Kg^−1^ day^−1^) or uvaol (UVA; 50 mg Kg^−1^ day^−1^) for two weeks. Left ventricular cross sectional area (LVCSA, A) and left ventricular wall thickness (LVWT, B) were measured in Masson’s trichrome stained sections. Two-three sections for each animal at the midregion area were analysed. Magnification 5X. Serum BNP levels were assessed by using a mouse BNP-specific ELISA (C). Values are mean ± SEM of 5–6 animals. *p<0.05 *vs* control. †p<0.05 vs angiotensin II.

In order to verify the potential antifibrotic effect of both triterpenes in vivo, we explored the effect of the administration of either erythrodiol or uvaol (50 mg Kg^−1^ day ^−1^) in mice infused with angiotensin II (1.44 mg Kg^−1^ day ^−1^, 2 weeks). As expected, angiotensin II led to significant cardiac hypertrophy at the organ and cellular level which was prevented by triterpenes treatment. Heart weight, normalized to body weight (HW/BW), was significantly increased in mice in response to angiotensin II when compared to vehicle-treated mice (Figure 7A). This hypertrophy seems to correlate with an increase in both cardiac myocyte size (Figures 7B, 7C, 7D), interstitial (Figures 8A, 8B, 8C) and perivascular matrix deposition (Figures 8F, 8G, 8H). These changes can participate in the left ventricle remodeling induced by angiotensin II since these animals show a higher LVCSA as well as LVWT as compared with controls (Figures 9A and 9B, respectively). Treatment with either erythrodiol or uvaol was able to reduce cardiac hypertrophy observed in angiotensin II-induced animals (Figures 7B, 7E and 7F, respectively) by decreasing both interstitial (Figures 8A, 8D and 8E, respectively) and perivascular fibrosis (Figure 8B, 8I and 8J, respectively). In addition, they also reduce the changes in both LVCSA and LVWT induced by angiotensin II (Figures 9A and 9B, respectively).

Next, the serum concentration of the BNP, a biomarker of cardiac damage, was measured. BNP is a cardiac neurohormone secreted from the cardiac ventricles as a response to ventricular volume expansion and pressure overload [33]. BNP levels have been shown to be elevated in patients with hypertrophic cardiomyophathy and left ventricular dysfunction, and correlated to New York Heart Association class as well as prognosis. As shown in Figure 9C, BNP levels were significantly higher in mice in response to angiotensin II when compared to vehicle-treated mice. Treatment with either erythrodiol or uvaol was able to reduce BNP levels observed in angiotensin II-induced animals (Figure 9C).

## Discussion

The study shows that the natural triterpenes, erythrodiol and uvaol, modulate some of the cardiac effects of angiotensin II. Indeed, the proliferation and fibrosis induced by angiotensin II in cardiac myofibroblasts *in vitro* was prevented by both triterpenes, as was the cardiac hypertrophy, left ventricle remodeling and fibrosis observed in angiotensin II infused animals. These actions could result in relevant benefits with potential clinical consequences, since activation of the renin-angiotensin system is a common feature of cardiovascular diseases [15].

Ample data have demonstrated that triterpenes from different origins exert antiproliferative actions on different tumoral cell lines [24], [34], [35]. Indeed, synthetic triterpenoids are found in phase I/II clinical trials for the treatment of leukemias and solid tumors [36]. The present data showed that erythrodiol and uvaol are also able to modulate proliferation and survival of non-tumoral cells. At low doses, both triterpenes were able to reduce angiotensin II-induced proliferation in cardiac myofibroblasts without affecting cell integrity and attachment through a PPAR-γ dependent pathway. However, at high doses erythrodiol and uvaol (25–50 µM) reduce cardiac myofibroblast survival both in the presence and absence of angiotensin II. Proliferation of cardiac myofibroblasts is an essential step involved in the reparative response to wound healing of damaged tissue. However, a large number of these cells, and especially hypersecretory myofibroblasts, can cause an aberrant remodeling through increased extracellular matrix deposition that favours functional alterations. Indeed, both triterpnes were able to reduce the cardiac remodeling induced by angiotensin II. Thus, our data suggest that triterpenes – besides exerting antitumoral actions – can produce beneficial effects on the cardiovascular system.

In agreement with previous studies [37], Ras/ERK1/2 seems to be the intracellular signaling pathway involved in the proliferative effect of angiotensin II in cardiac myofibroblasts. This is suggested by the fact that angiotensin II induced ERK1/2 phosphorylation, and that the presence of MEK inhibitor reduced this mitogenic effect. However, the modulation elicited by both triterpenes seems to be partially independent of ERK1/2 signaling. This assertion is supported by the fact that neither erythrodiol nor uvaol was able to modify the phosphorylation induced by angiotensin II at the dose which inhibits proliferation, which was only abrogated at the highest doses of the triterpenes (25–50 µM). In addition, the simultaneous presence of the MEK inhibitor and either erythrodiol or uvaol further reduces the proliferative activity of angiotensin II. Nevertheless, this inhibition also appears to be unrelated to their pro-apoptotic actions, since the pharmacological inhibition of ERK did not trigger any relevant increase in annexin-V binding in angiotensin II-stimulated myofibroblasts.

Various actions have been described by triterpenes in a PPAR-γ-dependent manner. It has been shown that some betulinic acid derivatives, among other triterpenes, act as PPAR-γ agonists, inducing differentiation of adipocytes [33]. Corosolic acid ameliorates obesity and hepatic steatosis in mice by increasing PPAR-γ expression in white adipose tissue [38]. The synthetic triterpenoid 2-cyano-3,12-dioxooleana-1,9-dien-28-oic acid increases cellular levels of PPAR-γ and regulates apoptosis in leukemic cells via caspase-8 [39]. Therefore, activation of PPAR-γ could be a pathway involved in the triterpene-exerted modulation of angiotensin II-proliferation. This hypothesis is confirmed by two facts: first, a specific PPAR-γ inhibitor is able to reverse the reduction induced by erythrodiol and uvaol on angiotensin II-induced proliferation and second, the reduced PPAR-γ nuclear levels induced by angiotensin II were abrogated upon treatment with the triterpenes. A modulatory role of PPAR-γ in the effects of angiotensin II in cardiac myofibroblasts has been previously reported. Administration of the PPAR-γ agonist rosiglitazone has been associated with a reduction in the proliferative effect induced by angiotensin II in murine cardiac fibroblasts [40], [41]. Likewise, chronic treatment with rosiglitazone to rats was able to partially prevent the collagen deposition induced by the infusion of angiotensin II [39]. Therefore, these data support that triterpenes might modulate the proliferative effect of angiotensin II in cardiac myofibroblasts by interfering with its effect on PPAR-γ.

A key molecule involved in cell survival is the stress kinase JNK, which has been found to mediate triterpene apoptosis in different tumoral cells [42]. Through pharmacological inhibition of JNK with SP600125, we have demonstrated that this pathway is a key component of the signalling involved in erythrodiol- and uvaol-induced apoptotic death in cardiac myofibroblasts. However, the inhibition of JNK pathway does not affect the antiproliferative response induced by low doses of triterpenes. It thus appears that JNK activation, while required for triterpene-induced apoptosis, is dispensable for their antimitogenic actions. We have also observed that these highest apoptotic doses of triterpenes cause retraction, rounding and shrinking of cardiac myofibroblasts and amorphous and condensed pattern. Cytoskeletal elements going to reorganization is a hallmark of apoptosis [43]. Similarly, we have previously observed that the apoptosis of astrocytoma induced by these triterpenes was accompanied by cytoskeletal protein rearrangements, as well as an altered expression of CD44, a molecule which facilitates cell-cell and cell-extracellular matrix communication [42].

We also found that erythrodiol and uvaol reduce the production of fibrosis in mice infused with angiotensin II. This can be a direct effect of triterpenes because a reduction in collagen I production was observed in triterpene-treated cardiac myofibroblasts. This thus suggests a modulatory role of these compounds of the profibrotic effect of angiotensin II. Extracellular matrix accumulation is a common response of the heart to different insults. However, as we have already mentioned, the excessive extracellular matrix deposition due to a large number of myofibroblasts can cause an aberrant remodeling through increased extracellular matrix deposition that favours functional alterations, since a reduced flexibility of heart can increase its filling pressure and contribute to diastolic dysfunction [7]. In fact, triterpenes were also able to prevent the left ventricular remodeling induced by angiotensin II in mice. This improvement was accompanied by a reduction in BNP levels, a biomarker of cardiac damage that is secreted from the cardiac ventricles as a response to ventricular volume expansion and pressure overload [33]. Therefore, these data suggest an improvement in cardiac function. Similarly, chronic or acute administration of triterpene-saponins to rats prevents the cardiac dysfunction and remodeling induced by diabetes or protects against myocardial ischemia-reperfusion injury [44], [45]. The administration of the triterpene lupeol also reduced the cardiac alterations associated with hypercholesterolemia [46]. Therefore, our results are in line with the observation that various triterpenes have demonstrated beneficial cardiac effects [27], [28].

In the last years, galectin 3 has emerged as an important mediator of cardiac remodeling in heart failure through its ability to stimulate fibrosis, although its specific role and the factors involved in its stimulation are under discussion. A recent study, supporting its profibrotic role, has established a relationship between serum galectin 3 and serum markers of cardiac extracellular matrix turnover in heart failure patients [47]. The present data show that angiotensin II is able to stimulate galectin 3 production in cardiac myofibroblasts, confirming previous data that reported an increase in galectin 3 levels in left ventricle of mice infused with a hypertensive dose of angiotensin II [48]. Moreover, in keeping with a preliminary report in which galectin 3 induced cardiac myofibroblast proliferation and collagen synthesis [49], our data might suggest a role of galectin 3 in the profibrotic effect induced by angiotensin II. This proposal is based on two facts: First, the increase in collagen I induced by angiotensin II paralleled an increase in protein levels of galectin 3; second, the decrease in collagen I levels in the presence of either triterpene was reflected with a reduction in galectin 3. This observation can be relevant from the clinical point of view because it has reported that galectin 3 levels are associated with adverse long-term cardiovascular outcomes in patient with heart failure [17].

Our in vivo findings confirm the potential cardiac effects of the natural triterpenes. Both inhibit left ventricle remodeling, hypertrophy and fibrosis in hearts from angiotensin II–infused mice. This antifibrotic effect could be a direct effect of these triterpenes because in cardiac myofibroblasts they restrain the production of collagen I and profibrotic mediator galectin 3. They also inhibit proliferation in cardiac myofibroblasts acting as PPAR-γ modulators. Given that pathological proliferation of cardiac myofibroblasts and the consequent extracellular matrix production are important contributors to the adverse cardiac remodeling that follows myocardial injury, our results suggest mechanisms by which erythrodiol and uvaol might exert beneficial effects on this process. In addition, these beneficial effects can be extended to other situations such as hypertension if we consider the important role of ventricular remodeling in the deterioration of cardiac function and the evolution to heart failure. Although we have reported potential signalling pathways that can be modulated by triterpenes, the study cannot clarify the effect of either erythrodiol or uvaol on the sequential activation or inhibition of the mentioned key signaling factors. However, the potential relevance of the triterpenes activity certainly deserves a further, but independent, study directed towards obtaining a better understanding of the downstream cascades and molecular mechanisms underlying their actions on cardiac fibroblast functions.
